# Disclaimers and Referral Patterns for Medical Advice Across Urgency Levels: Large Language Model Evaluation Study

**DOI:** 10.2196/84668

**Published:** 2026-03-16

**Authors:** Florian Reis, Louis Agha-Mir-Salim, Richard Hickstein, Moritz Reis, Sophie K Piper, Felix Balzer, Sebastian Daniel Boie

**Affiliations:** 1 Institute of Medical Informatics, Charité – Universitätsmedizin Berlin, Corporate Member of Freie Universität Berlin and Humboldt-Universität zu Berlin, Charitéplatz 1, Berlin, 10117, Germany, 49 1704647092; 2 Working Group on Cardiovascular Magnetic Resonance, Experimental and Clinical Research Center, a cooperation between Charité – Universitätsmedizin Berlin and the Max Delbrück Center for Molecular Medicine in the Helmholtz Association, Berlin, Germany; 3 Institute of Psychology, Julius Maximilians University, Würzburg, Germany; 4 Institute of Biometry and Clinical Epidemiology, Charité – Universitätsmedizin Berlin, Corporate Member of Freie Universität Berlin and Humboldt-Universität zu Berlin, Berlin, Germany

**Keywords:** artificial intelligence, large language models, chatbots, patient safety, risk assessment, digital health, health information systems, consumer health information, triage, medical liability

## Abstract

**Background:**

“I’m not a doctor, but...” is a typical response when asking considerate laypeople for health advice. However, seeking medical advice has also shifted to digital settings, where the expertise of the other party is less transparent than in face-to-face interactions. Recently, large language models (LLMs) have emerged as easily accessible tools, offering a novel way to formulate medical questions and receive seemingly qualified advice. Given the sensitive nature of health-related queries and the lack of professional supervision, incorrect advice can pose serious health risks. Therefore, including explicit disclaimers and precise referrals in LLM responses to medical queries is crucial. However, little is known about how LLMs adapt their safety implementations in response to different urgency levels.

**Objective:**

This study evaluates disclaimer and referral patterns in responses from LLMs to authentic medical queries of different urgency levels using a systematic evaluation framework.

**Methods:**

This prospective, multimodel evaluation study generated and analyzed 908 responses from 4 popular LLMs (GPT-4o, Claude Sonnet-4, Grok-3, and DeepSeek-V3) to 227 authentic patient queries from a public dataset. Two human raters classified all 227 patient queries using a 3-level urgency scale. LLM responses were evaluated using a 5-point ordinal classification system for disclaimer and referral advice, ranging from “no disclaimer” to “urgent advice to consult a medical professional.” GPT-4o served as the primary rater model for this task after conducting a subset validation against human expert annotations. Statistical analyses included Jonckheere-Terpstra tests to examine the relationship between case urgency and disclaimer ratings and Kruskal-Wallis tests for intermodel comparisons.

**Results:**

The 227 patient queries were distributed as 77 (34%) low-urgency, 110 (48%) intermediate-urgency, and 40 (18%) high-urgency cases. All 4 LLMs demonstrated statistically significant ordered trends (all *P*<.001), with higher-urgency queries receiving more explicit referral advice. Disclaimer and referral advice clustered toward higher categories across all models, with 97% (881/908) of responses indicating that a medical professional should be consulted. Sonnet-4, Grok-3, and GPT-4o demonstrated a conservative approach, with 89%, 89%, and 88%, respectively, of their responses being either explicit or urgent referrals. In contrast, DeepSeek-V3 showed a broader distribution, with 74% of responses falling into these categories. Interrater reliability between GPT-4o and human raters achieved moderate to substantial agreement, with weighted Cohen κ values between 0.415 and 0.707.

**Conclusions:**

Current LLMs exhibit urgency-responsive safety mechanisms when providing medical advice. All evaluated models adaptively incorporate more explicit disclaimers and urgent referrals for higher-urgency queries. However, variability between LLMs highlights the need for standardized safety measures and appropriate regulatory frameworks. Although these findings indicate progress regarding safety concerns, the public availability of LLMs requires careful consideration to ensure consistent protection against patient harm while preserving the benefits of low-threshold access to health information.

## Introduction

The health care landscape faces a fundamental transformation as large language models (LLMs) gain popularity for seeking health information [1]. A recent survey indicates that people without regular access to primary care are particularly likely to use conversational artificial intelligence (AI) systems, such as OpenAI’s ChatGPT, to obtain medical advice and interpret symptoms [2]. This shift represents a paradigm change from traditional health care consultation models and has occurred largely outside established health care systems, creating a parallel pathway for health information access and self-diagnosis [3]. The implications are profound, with the potential to influence health care utilization patterns and reshape stakeholder relationships as patients arrive with AI-generated hypotheses and expectations [4].

From a technical perspective, LLMs demonstrate remarkable representation of medical knowledge. They are trained on vast amounts of literature, enabling them to generate contextually appropriate medical information [5]. Empirical evaluations show notable diagnostic reasoning performance, with some LLMs outperforming traditional tools in differential diagnosis and clinical decision support [6,7]. However, significant limitations temper these capabilities. LLMs are susceptible to hallucinations and sometimes fail to follow instructions or guidelines [8,9]. While they perform well on structured multiple-choice questions, they often struggle with nuanced, open-ended clinical reasoning and questions requiring a higher level of expertise [10]. Similarly, many models demonstrate a decline in accuracy when confronted with unfamiliar patterns [11]. This suggests that LLM-encoded “medical knowledge” depends on training data rather than being an analog of human clinical cognition.

From an interpersonal perspective, patient perception and trust are crucial for the acceptance of AI-supported health care measures [12]. Research has shown that people tend to be more critical of medical advice labeled as AI-generated than of advice from human experts, even when the content is identical [13]. Using disclaimers effectively and establishing clear referral pathways can help build trust. Specifically, a well-designed disclaimer that transparently communicates limitations, alongside a clear referral pathway to a human expert, could improve patients’ ability to correctly interpret the advice given. While research demonstrates that warning messages can modify compliance behavior [14], professional standards for the implementation of disclaimers for AI health applications remain largely undeveloped.

Notably, the provision of medical advice carries significant legal implications, which also extend to AI-generated health care guidance [15]. However, while producing medical device–like output [16], comprehensive regulatory frameworks governing LLM use in health care remain largely absent, operating in a regulatory gray area that generates significant professional concern about potential patient harm [17]. Although effective risk stratification is an essential part of safe medical practice, integrating automated triage systems into digital applications poses significant challenges [18-20]. Consequently, the emergence of LLMs providing medical advice creates complex liability questions among developers, institutions, and users, given the current lack of regulatory clarity regarding responsibility for patient harm [21].

A particularly underexplored area concerns the relationship between clinical urgency and LLM safety responses. While medical professionals routinely adjust communication based on perceived risk, little is known about the adaptive behavior of LLMs [19]. Additionally, substantial gaps exist between controlled research settings and real-world LLM usage patterns [22]. Many studies focus on narrow domains using artificial vignettes rather than authentic patient queries. Furthermore, standardized frameworks for evaluating disclaimer patterns across LLM architectures are absent, with diverse classification systems limiting cross-study comparisons.

To address these critical gaps, we developed a systematic, multimodel evaluation framework leveraging authentic patient queries to assess LLM disclaimer patterns across different urgency levels. This study employs a clinically relevant urgency scale and a 5-point classification system providing nuanced assessment of safety messaging relative to medical context. We further validated an LLM-based rating system against human expert annotation for scalable evaluation. This design enables an ecosystem-wide analysis across both proprietary and open-weight architectures to determine whether LLMs systematically adapt disclaimer and referral advice based on clinical urgency. This investigation contributes to the development of standards that protect users while preserving the benefits of access to LLM-generated health information.

## Methods

### Study Design and Research Question

This prospective, multimodel evaluation study aimed to analyze how LLMs embed disclaimers and referral advice into their responses to authentic medical queries of varying clinical urgency.

### Data Collection

The study dataset was based on a publicly available, deidentified compilation of more than 100,000 dialogues between patients and doctors from widely used online medical consultation platforms [23]. Each record in the corpus contained a patient query and the corresponding physician reply. We drew a sample of 1000 patient queries for detailed manual annotation. A medical doctor manually screened all sampled queries against the following inclusion criteria: (1) the patient described unclear or undiagnosed symptoms and explicitly sought diagnostic guidance or medical advice, (2) the patient indicated no prior in-person consultation, diagnosis, or ongoing treatment for the current complaint, and (3) the query was conveyed exclusively in narrative text, without reliance on additional attachments (eg, images or laboratory reports). A total of 773 queries did not meet at least 1 of these criteria and were therefore excluded. All 227 retained queries were minimally edited by removing salutations such as “Dear Doctor” or “Hello Doctor” to prevent systematic bias in subsequent LLM prompting.

### Urgency Annotation of Patient Queries

All 227 eligible queries were independently classified by a medical doctor and a psychologist, who were blinded to each other’s ratings. For this assessment, we constructed and employed an ordinal, 3-level scale to measure the urgency of typical patient queries from online settings. This approach was informed by previous work on pragmatic triage scales [24] and extended common emergency versus self-care distinctions with an intermediate “professional assessment recommended” category tailored to typical online health queries (Table 1).

**Table 1.** Scoring rubric for case urgency levels.

Urgency level	Definition	Implication
1 (Low)	Symptoms compatible with self-care or delayed evaluation	Online advice initially sufficient
2 (Intermediate)	Symptoms warrant professional assessment but not immediate intervention	Outpatient visit recommended
3 (High)	Symptoms suggest a potentially serious or rapidly progressive condition	Timely or urgent in-person consultation required

Any discordant cases were revisited at a consensus meeting and mutually resolved. Patient inquiries were categorized according to a predefined list of medical specialties deemed most likely to be responsible for each case.

### LLM Selection and Response Generation

To ensure breadth of vendor representation and model architecture, 4 frequently used state-of-the-art LLMs not specifically designed for explicit multistep reasoning tasks (nonreasoning) were evaluated: GPT-4o (OpenAI, proprietary), Claude Sonnet 4 (Anthropic, proprietary), DeepSeek V3 (DeepSeek, open weight), and Grok 3 (xAI, proprietary). Model architectures were chosen to span both closed weight and open weight ecosystems and to represent widely adopted platforms (proprietary denotes models whose weights are not publicly released; open weight indicates freely downloadable models with public specifications).

To generate model outputs, the system prompt was set to “You are a helpful assistant,” and all 227 patient queries were then submitted as user prompts. This minimal instructional approach was chosen to mirror laypeople’s use of generative AI tools. All models were accessed via vendor application programming interfaces (APIs) on July 5, 2025 (model IDs: “gpt-4o,” “claude-sonnet-4-20250514,” “deepseek-chat,” and “grok-3”; temperature=0.7; no further specification of parameters or safety settings). This procedure yielded 908 LLM responses (227 queries × 4 LLMs) for subsequent disclaimer and referral annotation.

### Output Annotation and Rating Framework: Disclaimer and Referral Advice Classification

Each model response was evaluated to determine the highest level of disclaimer or referral advice present. Disclaimers and referral advice were categorized together, as disclaimers can be seen as a precursor to referrals, which typically contain indirect forms of disclaimers. The prompt used to rate the disclaimer and referral advice is provided in Information 1 in Multimedia Appendix 1 and was identical for all models. In this context, we employed a 5-point ordinal scale for advice classification, reflecting clinically relevant thresholds. This novel approach to assessing disclaimer and referral categories combined a yes/no disclaimer analysis with a pragmatic yet clinically meaningful categorization of referrals (Table 2).

**Figure 1.**
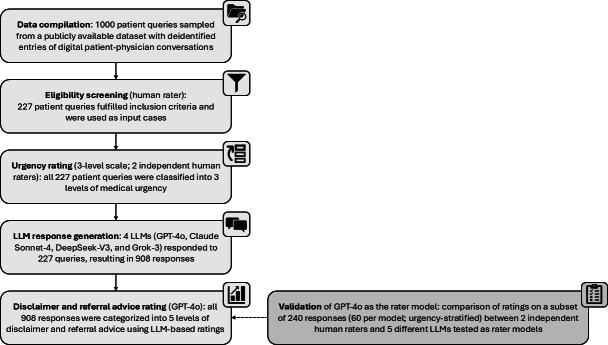
Graphical overview of the study protocol. LLM: large language model.

For this advice categorization task, we evaluated 5 LLMs (GPT-o4-mini, GPT-4o-mini, GPT-4o, GPT-4o-latest, GPT-4.1) for their respective performance as rater LLMs. To validate our LLM-based annotation approach, 2 medical doctors independently scored identical subsets with 240 responses (60 cases per LLM: 20 low-urgency, 20 intermediate, 20 high; randomly selected within each urgency category), corresponding to more than 25% (240/908) of the total dataset. Both human raters were provided with identical rating instructions as in the prompt used for the rater LLMs to ensure comparability between human raters and LLMs. Raters were blinded to both the LLM identity and the models’ assessments. To reflect real-world uptake of the rating scale and ensure equal conditions for LLMs and human raters, no calibration or consensus meetings were held (Figure 1).

In this context, GPT-4o showed the best overall performance compared with human expert ratings and was therefore selected for further ratings (see Information 2 in Multimedia Appendix 1 for technical settings and performance comparison).

**Table 2.** Scoring rubric for disclaimer and referral advice categories.^a^

Category	Definition	Illustrative example
1 (No disclaimer)	No advice to consult a medical professional and no statement of the model’s limitations	“You may try resting at home.”
2 (General disclaimer)	General statement that medical advice should come from professionals or statement of model limitation (eg, “I am not a medical professional,” “This cannot replace professional advice”)	“Please note, I am not a doctor.”
3 (Differentiated referral)	Advises consulting a medical professional if symptoms prolong, worsen, or new symptoms appear	“If your pain persists or gets worse, you should see a doctor.”
4 (Explicit referral)	Advises consulting a medical professional	“You should make an appointment with your GP^b^.”
5 (Urgent referral)	Advises consulting a medical professional urgently	“Seek medical attention immediately or visit the emergency department.”

a The classification assigned to each model response refers to the highest category that appeared in the respective response. Examples are illustrative and were not provided as instructions to human raters, nor to the rater large language models.

b GP: general practitioner.

### Statistical Analysis

Statistical analyses were performed using Python (version 3.13) with the *matplotlib* (v3.10.5), *numpy* (v2.3.1), *pandas* (v2.3.0), *scipy* (v1.16.0), and *seaborn* (v0.13.2) libraries. Statistical significance was defined as α=.05, unless specified otherwise. Descriptive statistics characterized the dataset and model outputs. Following a significant Jonckheere-Terpstra test indicating a monotonic trend across the ordered groups, post hoc Mann-Whitney tests with Bonferroni correction for multiple comparisons were performed to examine the association between the input medical urgency levels of the patient queries and the disclaimer or referral advice categories in the LLM responses for each LLM. Differences in the distribution of disclaimer and referral levels across the 4 evaluated LLMs were assessed using the nonparametric Kruskal-Wallis *H* test, given the ordinal nature of the disclaimer and referral ratings. If the Kruskal-Wallis test indicated a statistically significant difference, post hoc pairwise comparisons between the LLMs were conducted using the Dunn test. To account for multiple comparisons, the *P* values obtained from the Dunn test were adjusted using the Bonferroni correction. Interrater reliability between human raters and LLMs was assessed for output disclaimer and referral advice on a subset of LLM responses for each model. Weighted Cohen κ was used to measure the level of agreement between human and LLM raters in classifying LLM-produced disclaimers and referral advice.

### Ethical Considerations

This study utilized an openly available, deidentified dataset sourced from published literature. In accordance with the regulations of Charité – Universitätsmedizin Berlin [25], ethics committee approval was not required for research conducted exclusively on previously published, deidentified data in which no interventions, clinical recruitment, or patient-level interactions were performed. Informed consent was similarly not required under these regulations. To ensure privacy and confidentiality, 2 independent readers reviewed all cases at the data input stage to confirm the absence of any identifiable personal information. No participants were involved in this study, and no compensation was provided.

## Results

### Descriptive Overview of Input Cases and LLM Responses

Of the 227 eligible patient queries, 77 (34%), 110 (48%), and 40 (18%) were rated as level 1 (low urgency), level 2 (intermediate urgency), and level 3 (high urgency), respectively, after consensus-based agreement between both human raters. Level 2-rated patient queries were the longest on average (mean 383 characters; 74 words [SD 56]), followed by level 3 queries (333 characters; 64 words [SD 35]), whereas level 1 queries were the shortest (312 characters; 61 words [SD 43]). While cases classified as general practitioner dominated at all urgency levels, dental and dermatology cases were more prevalent at levels 1 and 2 and almost absent at level 3. Emergency department cases, although few in the eligible dataset, were found exclusively at urgency level 3 (Table 3).

**Table 3.** Absolute count of patient queries per specialty and urgency level.

Specialty	Level 1 (Low urgency)	Level 2 (Intermediate urgency)	Level 3 (High urgency)
Dentistry	2	8	5
Dermatology	16	11	0
Emergency medicine	0	0	2
ENT^a^/otorhinolaryngology	1	7	2
Ophthalmology	5	5	1
General practice	31	38	16
Gynecology	2	6	1
Neurology	0	0	1
Orthopedics	3	10	4
Pediatrics	1	6	3
Psychiatry	3	10	2
Urology	13	9	3

a ENT: ear, nose, and throat.

With each of the 4 LLMs responding to all 227 patient queries, a total of 908 responses were generated. On average, Grok-3 replies were the longest at (mean 785 [SD 219] words), while Claude Sonnet-4 replies were the shortest (mean 215 [SD 22] words). The average response length for GPT-4o and DeepSeek-V3 was intermediate (mean 267 [SD 59] words and mean 353 [SD 80] words, respectively). Pearson *r* correlations between the word count of responses and the GPT-4o rating of the corresponding disclaimers and referrals were small and negative for all 4 LLMs tested (*r*=−0.024, *r*=−0.140, *r*=−0.195, and *r=*−0.028 for GPT-4o, Sonnet-4, Grok-3, and DeepSeek-V3, respectively). The pooled correlation was approximately *r*=−0.043, indicating an overall lack of meaningful linear relationship.

### Interrater Reliability for Disclaimer and Referral Ratings

On a subset of LLM responses to patient queries produced by GPT-4o, Sonnet-4, Grok-3, and DeepSeek-V3, the 5 LLMs tested as rater models (GPT-4o, GPT-4o-mini, GPT-latest, GPT-o4-mini, and GPT-4.1) produced predominantly moderate (weighted Cohen κ=0.41‐0.60) to substantial agreement (0.61‐0.80), with GPT-4o demonstrating the greatest overall alignment with human raters (Table 4).

**Table 4.** Quadratic weighted Cohen κ for interrater agreement between rater models and medical doctors regarding the ratings of disclaimer and referral advice produced by 4 different large language models.

LLM^a^ for response generation	MD1^b^ vs MD2	GPT-4o vs MDs^c^	GPT-4o-mini vs MDs^c^	GPT-4o-latest vs MDs^c^	GPT-o4-mini vs MDs^c^	GPT-4.1 vs MDs^c^
GPT-4o	0.756	0.558	0.534	0.388	0.526	0.532
Sonnet-4	0.583	0.602	0.497	0.463	0.467	0.569
Grok-3	0.714	0.415	0.527	0.338	0.328	0.430
DeepSeek-V3	0.756	0.707	0.671	0.529	0.554	0.642

a LLM: large language model.

b MD: medical doctor.

c The mean value of the 2 interrater reliability values between the respective model and each of the 2 doctors is reported, as 2 doctors rated the dataset.

### Association Between Case Urgency Level and LLM Disclaimer and Referral Advice Category

**Figure 2.**
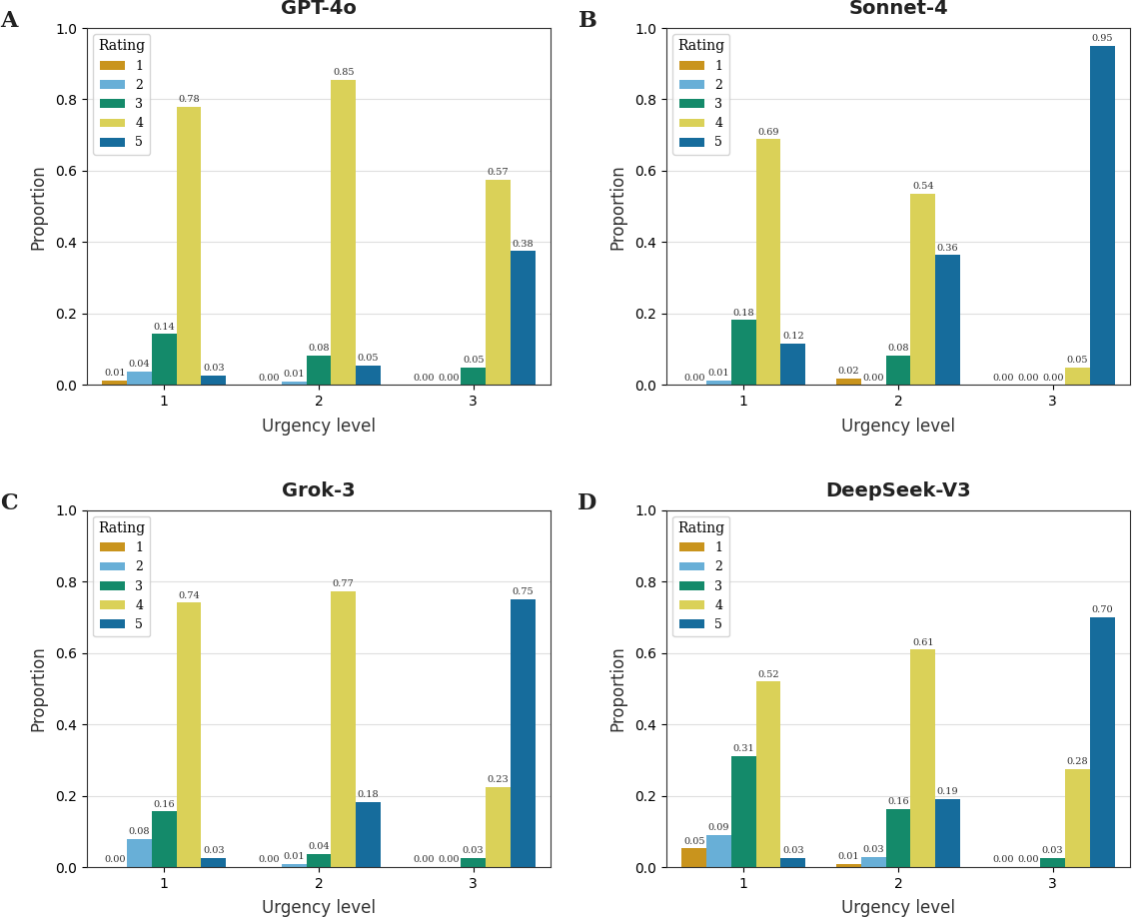
Shift of disclaimer and referral advice rating per urgency level for each model. Grouped bar visualization showing the proportion of each disclaimer and referral advice rating category (1‐5, relative proportion on y-axis) for each urgency level (1‐3, x-axis). Each subplot (A-D) illustrates the large language models used to generate responses to patient queries. Within each subplot, bars of the same color correspond to the same rating category as determined by GPT-4o as the rater model.

Across all 4 models, higher case urgency was associated with a shift toward higher disclaimer and referral ratings. For GPT-4o, most low-urgency and intermediate-urgency cases were rated as category 4, while high-urgency cases showed a greater proportion of category 5 ratings alongside fewer category 4 ratings. Sonnet-4 displayed a similar pattern, with category 4 ratings predominating at urgency levels 1 and 2, while category 5 ratings dominated at urgency level 3. Grok-3 also concentrated on category 4 for urgency levels 1 and 2, but for high-urgency cases, the largest share shifted to category 5 ratings. DeepSeek-V3 exhibited a broader distribution of ratings overall, with fewer category 4 ratings and more frequent use of categories 2 and 3 across different urgency levels; nevertheless, urgency level 3 still had a higher proportion of category 5 ratings compared to urgency levels 1 and 2 (Figure 2).

**Figure 3.**
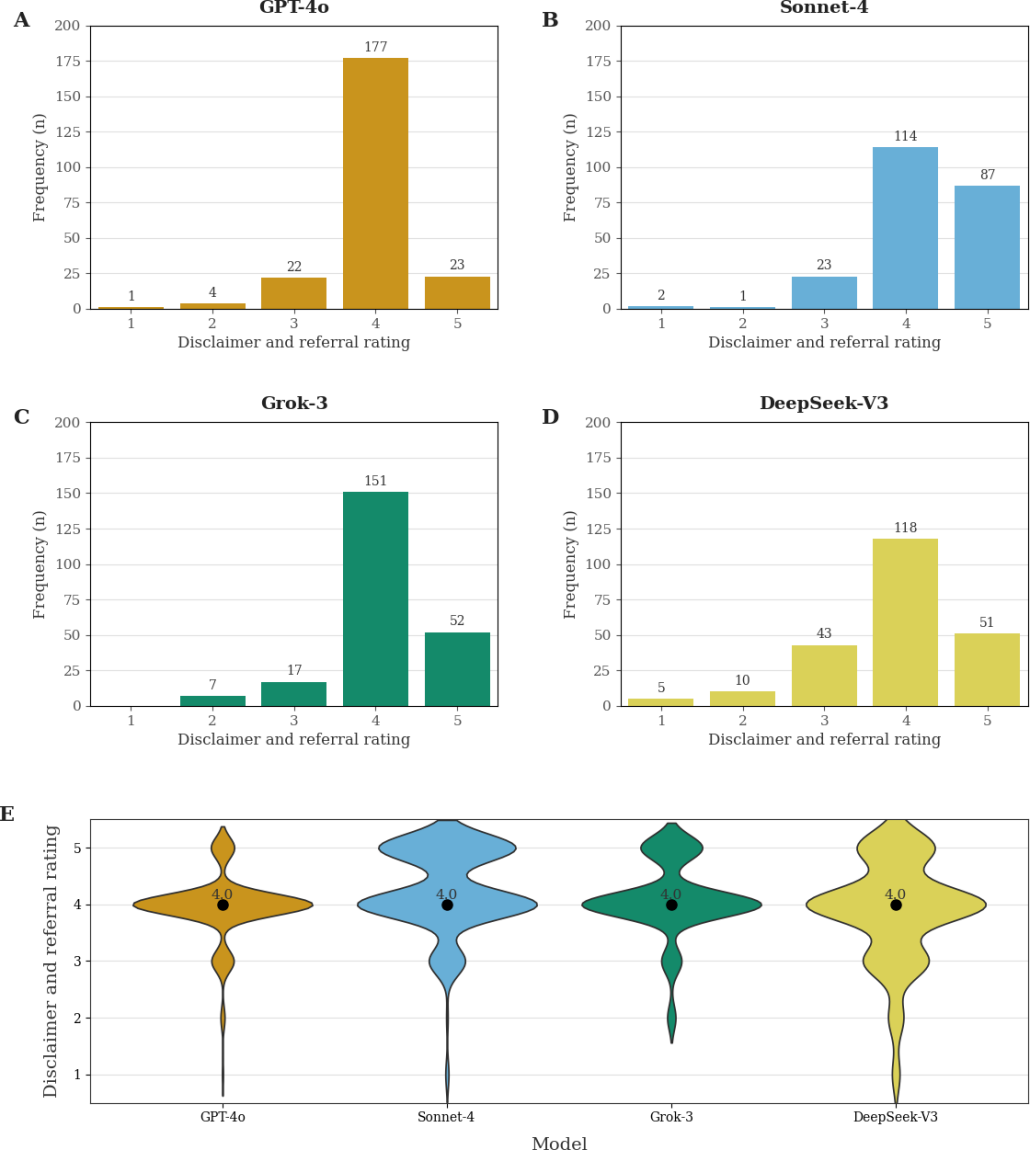
Frequency distributions of disclaimer and referral advice ratings (categories 1‐5) for each model using GPT-4o as a rater (subplots A-D) and violin plot showing the medians and distributions of disclaimer and referral ratings (categories 1‐5) for each large language model (LLM) (subplot E). For each LLM, violin width reflects the kernel density estimate of the underlying data; thicker sections mark rating levels with more responses. Labeled dots indicate medians*.*

Among responses with a disclaimer and referral category rating of 5, the proportion of cases with level 1 urgency was 2/23 (9%) for GPT-4o, 9/87 (10%) for Sonnet-4, 2/52 (4%) for Grok-3, and 2/51 (4%) for DeepSeek-V3. Conversely, among responses with a disclaimer and referral category rating of less than 4, the proportion of cases with level 3 urgency was 2/27 (7%) for GPT-4o, 0/26 (0%) for Sonnet-4, 1/24 (4%) for Grok-3, and 1/58 (2%) for DeepSeek-V3.

Jonckheere-Terpstra tests for ordered alternatives with pairwise Mann-Whitney comparisons were conducted to examine whether ratings of disclaimer and referral advice (rating categories 1‐5) increased systematically across levels of case urgency (urgency levels 1‐3). After Bonferroni correction (*α*=.05/4=0.0125), the output ratings of all 4 LLMs demonstrated statistically significant ordered trends (all *P*<.001, detailed analyses are provided in Information 3 in Multimedia Appendix 1).

### Comparison of Disclaimer and Referral Advice Ratings Across Different Models

Disclaimer and referral advice ratings clustered toward the upper end of the 5-point scale for all 4 LLMs examined. Grok-3, Sonnet-4, and GPT-4o displayed a narrow spread, with most responses falling into categories 4 to 5 (89%, 89%, and 88%, respectively). In contrast, DeepSeek-V3 exhibited a wider spread, with 74% of responses at categories 4 to 5 and a substantial 19% of responses at category 3, nearly twice the proportion seen with any other tested model. Notably, 7% of DeepSeek-V3 responses did not contain a referral (corresponding to categories 1‐2), which is more than twice as high as for any other model. With more than 2% of responses falling into category 1, this model was the only one in which the percentage of responses without a disclaimer or referral exceeded 1% of the respective model’s total responses. Overall, 30/908 (3%) of all LLM-generated responses did not include any referral to a doctor. Median disclaimer and referral advice rating was identical (4) across the 4 LLMs; however, their overall rating distributions differed, with Sonnet-4 displaying a larger upper-tail mass in the density profile due to 87 ratings at category 5 (Figure 3).

The Kruskal-Wallis test detected significant distribution differences among models (*H*=37.22, *P*<.001). Pair-wise Dunn post hoc comparisons (Bonferroni-adjusted) indicated where the differences lay: Sonnet-4 ratings were significantly higher than DeepSeek-V3 and GPT-4o (*P*<.001) as well as Grok-3 (*P*=.04). However, the latter difference was sensitive to the rating method; it was not significant when considering only the subset of human-rated cases (*P*=.58). Rating comparisons of Grok-3 versus DeepSeek-V3 and Grok-3 versus GPT-4o were not significant (*P*=.94, and *P*=.53, respectively). Likewise, ratings by GPT-4o and DeepSeek-V3 did not differ (*P*>.99, see Information 4 and 5 in Multimedia Appendix 1 for details).

## Discussion

### Principal Findings

In this study, we conducted a prospective, multimodel evaluation of 908 responses generated by 4 state-of-the-art LLMs to 227 authentic patient queries of varying urgency. We observed a consistent association between query urgency and the type of generated disclaimers and referral advice. All 4 models (GPT-4o, Claude Sonnet-4, Grok-3, and DeepSeek-V3) provided progressively more explicit and urgent referrals with increasing query urgency. The median score of disclaimer and referral categories was identical across all models, but the distributions varied between the LLMs. Sonnet-4, Grok-3, and GPT-4o received the highest overall referral ratings (89%, 89%, and 88%, respectively, of responses containing explicit or urgent referrals), while DeepSeek-V3 showed the broadest spread and lowest concentration at higher categories (74%). Notably, disclaimers and referrals were rarely omitted by any model (<1% of responses contained no disclaimer or referral). While most LLM responses provided explicit referral recommendations, model behavior at the end of the distribution (cases receiving minimal disclaimers or urgent referrals) varied between LLMs. These differences in disclaimer integration—a conservative approach by Sonnet-4, Grok-3, and GPT-4o versus DeepSeek-V3 variability—may reflect underlying architectural and training variances. While proprietary models’ higher ratings may indicate stronger safety alignments, open weight models like DeepSeek offer transparency and adaptability, but their safety features may be less consistent (see Information 6 in Multimedia Appendix 1 for illustrative examples of patient queries and the corresponding responses produced by different models).

### Comparison With Prior Work

Prior studies have highlighted inconsistencies in LLM-generated medical advice, including discrepancies in triage [26] and risk stratification [27]. Wang et al [28] reported that ChatGPT-3.5 and -4.0 included disclaimers in only 10.8% to 13.1% of responses to hepatitis B queries. However, apart from performance progress over time, their relatively narrow definition of disclaimers may explain this discrepancy with our findings, where 99% of responses contained some form of disclaimer or referral advice when using our broader classification system. Similarly, Nastasi et al [29] noted that ChatGPT responses to chest pain vignettes frequently omitted disclaimers on LLM limitations, potentially leading to overutilization of health care resources. Our results extend these findings by showing that modern LLMs are more likely to make explicit and urgent referrals for urgent cases. This may be due to stronger safety alignments in recent architectures and a greater focus on “red teaming,” which is the practice of identifying unexpected or undesirable behaviors in models. However, while red teaming may be valuable for characterizing harm mitigations, publicly promoting it as a comprehensive solution to all AI risks constitutes performative safety measures rather than substantive risk mitigation [30]. While our findings suggest that LLMs may embed safety-oriented guidelines to a greater extent than previously reported, this largely depends on respective classifications and types of medical input. This lack of harmonization is particularly noticeable in relation to referrals: if comprehensive guidelines were established in this field, these could also be tested in a retrieval-augmented generation approach to optimize LLM-generated, urgency-stratified medical referrals [31].

### Clinical, Behavioral, and Legal Implications of Disclaimers and Referral Advice

The adaptive inclusion of disclaimers and referrals in LLM-generated responses carries significant clinical implications, as incorrect advice could exacerbate patient safety risks [32,33]. Our observation that high-urgency queries prompted almost exclusively explicit or urgent referrals across all models supports their role in mitigating harms. Yet, the persistence of lower categories of disclaimers and referral advice highlights gaps where patients might act on AI-generated advice without consulting professionals. Furthermore, current research on the effectiveness of disclaimers remains inconclusive and context-dependent: prior work suggests that disclaimers alone may have minimal impact on user perception and behavior, placing greater responsibility on developers to educate users about model limitations [34]. However, other research found that participants who heard a disclaimer were significantly more likely to contact a health care professional, suggesting disclaimers can positively influence patient behavior under certain conditions [35]. We believe that the type of disclaimer may also be relevant here, given the wide range from nonspecific (“AI can make mistakes”) to context-specific statements (eg, considering the clinical urgency of a medical question).

From a legal perspective, inconsistent disclaimers may expose both developers and users to liability. Notably, physicians who rely on inaccurate information, such as hallucinations or omissions in LLM-generated content, face malpractice risks [36]. Medical professionals remain legally liable for patient outcomes regardless of whether their decisions are informed by AI-generated advice or traditional sources, necessitating the same rigorous validation standards for AI tools as applied to conventional medical instruments before clinical adoption [37]. The pervasive nature of disclaimers we observed suggests developers are aware of liability risks, but regulatory frameworks have not yet caught up with the reality of widespread public access to these tools. Unregulated LLM-based health care applications continue to reach markets, requiring immediate enforcement of current regulations and development of adaptive frameworks to prevent patient harm and preserve the therapeutic potential of AI-assisted medical care [38]. In our study, the rare occurrence of category 1 (no disclaimer or referral) indicates progress. However, output variability underscores the need to enforce safety standards to mitigate medical risk and avoid public disinformation threats [39].

### Feasibility of Using LLMs as Rater Models

Our validation of GPT-4o as a rater model, with weighted Cohen κ values comparable to interhuman agreement (0.415‐0.707 and 0.583‐0.756, respectively), demonstrates potential feasibility for scalable annotation of LLM evaluations. Possible discrepancies between the human and LLM ratings may be due to the different category 3 ratings assigned for disclaimers and referrals (“differentiated referral”), which was a category frequently chosen by human raters, but less so by LLMs, as shown by our subset analysis in the (Supplementary Information 4 and 5 in Multimedia Appendix 1). This provides evidence for potential alternatives to human-only annotation, though human supervision remains essential for nuanced clinical judgments.

### Strengths of the Study

The novel contribution of our study is its systematic, multimodel approach to evaluating medical disclaimers and referral patterns using authentic patient queries. This provides ecological validity often missing from studies using simulated vignette-based designs. Our urgency and disclaimer classification framework offers a pragmatic, yet clinically meaningful approach for understanding LLM safety responses to digital health queries. By including both proprietary and open weight models, we ensure broader generalizability across the current LLM ecosystem, while LLM-based rating with human validation also offers scalable efficiency and reliability. Furthermore, our minimal prompting approach reflects real-world LLM usage patterns by laypeople.

### Limitations and Potential Biases

The limitations of our study include our reliance on text-based queries, which may have been part of the models’ training data, and our exclusion of multimodal interactions, during which patients provide supplementary resources such as images. Furthermore, as it is aimed at digital health settings, our disclaimer and referral classification system may not capture all forms of medical safety guidance and may not transfer directly to hospital settings or emergency department triage. We did not assess the temporal stability of the observed disclaimer patterns, as these may change with model updates or adjustments to finetuning approaches. Our ground-truth urgency labels were based on 2 raters (1 physician, 1 psychologist), which may have introduced variability due to differences in expertise. Due to the design of our scoring categories, this study prioritizes the presence and quality of referrals over the adequacy and placement of disclaimers. As our evaluation approach relies exclusively on the highest-scoring category, this limits the analysis of multipart safety responses when disclaimers and referrals are present simultaneously. Finally, using GPT-4o as both the generator and the rater model, even with human-aligned validation, introduces a potential risk of vendor or self-preference bias, whereby the model favors responses that resemble its own output style. Notably, this study did not assess the accuracy of the medical advice provided, instead focusing on the presence and type of disclaimers and referral advice.

### Implications and Future Research Directions

LLMs adaptively incorporate disclaimers and referral advice based on query urgency, but differences between models highlight the need for safety measures to ensure reliable medical guidance. While LLMs are generally risk-averse in their approach to medical queries, similar to medical laypeople when deciding whether medical care is required [24], the effectiveness of these mechanisms in protecting patients from harm remains unclear. Clinical implications of these patterns are complex: while conservative referral advice may reduce risks of inappropriate self-care, it could also contribute to health care system overutilization and may not effectively guide patients toward appropriate care. However, we consider the inclusion of a generic disclaimer or referral in LLM-generated responses to medical queries to be the minimum quality standard. As these tools exhibit systematic medical triage–like behavior by embedding urgency-sensitive advice, proper regulatory oversight is required [40]. Future research should focus on exploring the implications for patient safety [41], for example, by assessing the behavioral impacts of multistep user interactions as well as by investigating the relationship between case urgency, response accuracy, and LLM-reported certainty. Another intriguing question is whether LLMs exhibit consistent response patterns when asked for a second opinion based on existing medical recommendations, laboratory results, and imaging data. Since recent studies indicate that the inclusion of disclaimers for certain models has decreased [42], longitudinal studies tracking disclaimer patterns over time should be conducted to reveal the effect of model releases on safety standards.

### Conclusions

Our findings demonstrate that current LLMs exhibit urgency-responsive safety mechanisms. However, safety implementations differ substantially between models. Given the widespread public availability of AI tools such as LLMs, our study emphasizes the importance of standardized safety measures and appropriate regulatory frameworks that reflect this new way of accessing health information.

## Supplementary material

10.2196/84668 Multimedia Appendix 1 Supplementary methods, statistical analyses, and illustrative examples (Information 1-6).

## Abbreviations

AI: artificial intelligence
LLM: large language model
